# Calpain Cleavage Prediction Using Multiple Kernel Learning

**DOI:** 10.1371/journal.pone.0019035

**Published:** 2011-05-03

**Authors:** David A. duVerle, Yasuko Ono, Hiroyuki Sorimachi, Hiroshi Mamitsuka

**Affiliations:** 1 Bioinformatics Center, Kyoto University, Uji, Kyoto, Japan; 2 Calpain Project, Rinshoken, Tokyo, Japan; Kyushu Institute of Technology, Japan

## Abstract

Calpain, an intracellular 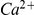-dependent cysteine protease, is known to play a role in a wide range of metabolic pathways through limited proteolysis of its substrates. However, only a limited number of these substrates are currently known, with the exact mechanism of substrate recognition and cleavage by calpain still largely unknown. While previous research has successfully applied standard machine-learning algorithms to accurately predict substrate cleavage by other similar types of proteases, their approach does not extend well to calpain, possibly due to its particular mode of proteolytic action and limited amount of experimental data. Through the use of Multiple Kernel Learning, a recent extension to the classic Support Vector Machine framework, we were able to train complex models based on rich, heterogeneous feature sets, leading to significantly improved prediction quality (6% over highest AUC score produced by state-of-the-art methods). In addition to producing a stronger machine-learning model for the prediction of calpain cleavage, we were able to highlight the importance and role of each feature of substrate sequences in defining specificity: primary sequence, secondary structure and solvent accessibility. Most notably, we showed there existed significant specificity differences across calpain sub-types, despite previous assumption to the contrary. Prediction accuracy was further successfully validated using, as an unbiased test set, mutated sequences of calpastatin (endogenous inhibitor of calpain) modified to no longer block calpain's proteolytic action. An online implementation of our prediction tool is available at http://calpain.org.

## Introduction

Calpain (EC 3.4.22.17, Clan CA, family C02) is an intracellular 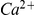-dependent cysteine protease known to regulate substrate functions by limited proteolysis, i.e. proteolytic processing [1]–[8], resulting in the modulation of a wide variety of biological phenomena. The many known homologues of calpain constitute a major protease family distributed over a wide range of organisms. Calpain has been associated with regulation of signal transduction system, cell motility and apoptosis, while malfunction has been observed in several serious diseases in human [3], including muscular dystrophies [9], [10], diabetes [11], [12] and tumorigenesis [13], [14].

For precise modulation of substrate functions by calpains, the cleavage sites are anticipated to be strictly determined depending on substrates [15]. In other words, the positions of the cleavage sites are essential determinants for how calpains modulate substrate functions. Therefore, prediction of cleavage sites by calpains is crucial to gain insight into how calpain proteolysis modulates cellular functions through substrate proteolysis [8]. The prediction holds an advantage when available amounts of substrates are low and cleavage site determination by protein chemistry such as protein sequencing and mass-spectrometry is impossible. If cleavage sites are determined, antibodies specific to the sites [16]–[18] and inhibitors for specific substrate proteolysis [19]–[21] can be designed to analyze proteolytic events by calpain under various conditions. Many studies have been attempted to predict calpain cleavage sites [22]–[24], however, precise prediction has never been successful so far.

Mechanisms of substrate recognition by calpain are altogether poorly understood, compared to other types of proteases. For example, while PEST motifs (sequences rich in proline, glutamic acid, serine and threonine) have been shown to play a role in calpain recognition for some substrates [25], numerous studies have also identified cases for which PEST motifs do not impact substrate recognition or cleavage [26], [27]. Attempts at predicting substrate cleavage by calpain have so far been entirely built on empirically derived rules for position-based residue preferences [19] and, more generally, Position-Specific Scoring Matrix methods [22], although the importance of higher order structure information has long been established [28].

A number of different methods [29]–[32] have been developed to predict substrate recognition and cleavage by proteases other than calpain, notably caspases: another family of cysteine protease involved mainly in apoptosis, as well as in various biological phenomena also involving calpain at times [33]. However, despite their similarity, calpain's particular mode of proteolytic action would appear to set it apart from caspase, and different methods seem needed in order to attain similar prediction results. Difficulties of predicting calpain cleavage sites probably originate from the structure and functions of calpains: calpains can proteolyze various substrates *in vitro* and *in vivo* that are involved in a variety of cellular processes [8]. To achieve this, substrate binding sites of calpain molecules may have evolved to recognize their substrates in a wide range of peptide sequences, rather than binding strongly to a few specific amino acid residues around cleavage sites in a fashion similar to trypsin or caspases (which have predominant K/R and D residue preferences at the P1 site, respectively [34]–[36]). As a consequence, elucidating the mechanisms of substrate cleavage by calpains, requires complex combinatorial analysis of a wide range of amino acid sequences around substrate cleavage sites.

Currently, CaMPDB, an online repository of calpain sequences [37], lists a little over a hundred confirmed substrate sequences, along with a computationally expanded set of many thousands potential substrate candidates, obtained through BLAST alignment search. While a crucial help to devise machine-learning cleavage prediction methods, the limited number of confirmed cleavage data, compounded by the presence of important selection biases in the set, further complicates the task of reaching prediction performances on calpain cleavage comparable to other types of cysteine protease (by contrast, in their recent work on caspase cleavage prediction, Song *et al.* [32] had access to data for 562 cleavage sites over 370 sequences).

Over the past twenty years, Support Vector Machine algorithms have become a ubiquitous tool in machine learning and occupy a prominent position in bioinformatics research. In addition to belonging to the margin-maximizer group of classifiers (thus providing a bound on the generalization error), SVM distinguish themselves by the use of so-called kernel functions to transform the input data before classification. Traditional SVM algorithms, such as used by recent related work on protease substrate prediction [32], [38] require selecting a single kernel function and using it on all input data throughout the algorithm. Such work emphasized the importance of using richer feature sets (such as secondary structure information in addition to sequence), however, because of the nature of standard single-kernel methods, had to compromise on the type and format of features that could be used.

The use of recent extensions to the SVM framework, commonly known as Multiple Kernel Learning (MKL) algorithms allowed us to combine heterogeneous feature sets, each with their own adapted kernel function, while optimizing the contribution of each sub-kernel to the resulting classifier.

Most interestingly, it has been shown [39] that Multiple Kernel Learning can give a good understanding of which feature sets are important for discrimination. While standard SVM methods produce classification function that are notoriously difficult to interpret in terms of feature contribution compared to other classification techniques, MKL yields weights for each sub-kernel that, once properly scaled, provide a useful representation of the relative discriminative power of each set of features.

## Materials and Methods

### Optimizing Feature Set Contribution through Multiple Kernel Learning

At the heart of kernel methods, the “kernel-trick” makes use of kernel functions to remap input data into a high dimensional feature space where a variety of methods can be used to efficiently analyze the data (e.g.: find a margin-maximizing separating hyperplane, in the case of SVM). The choice of such a kernel function not only affects separability of data in the feature space, but can also help efficiently filtering in or out certain characteristics of the input without the need for additional steps.

A kernel function does not explicitly calculate data coordinates in the feature space, but instead computes the inner products between the images of all pairs of input vectors in that space.

Given a kernel function, 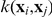, and a set of labeled training instances 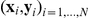 (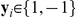), training an SVM means learning the weights (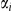) in the decision function:
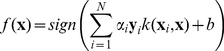
(1)Where 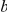 is the bias.

Judicious choice of kernel function (see below) gives great flexibility regarding the nature of features that can be used (real values, binary values, strings…), but it can sometimes be desirable to combine features of different structure or dimension within the same classifier. In such case, a standard solution is to find a common encoding that can be satisfyingly applied to each set of features in order to produce a unique input vector for each instance. Going with such an approach, however, means losing potentially useful data structure information in the encoding and being forced to use identical kernel parameters for all data sources. Additionally, it is very difficult to extract useful information in terms of feature contribution to the final classifier.

A more elegant solution resides in the use of “multiple kernel learning”. Although there exist a variety of methods [39]–[41], they all tend to rely on expressing a combined kernel as a linear sum (2) of 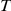 sub-kernel functions (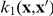
… 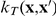), leading to the decision function (3) and its associated optimization problem.
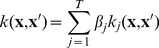
(2)

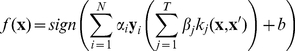
(3)


[39] offers a method to reformulate the problem as a “semi-infinite” linear program, that can in turn be solved using standard LP techniques.

### Selecting Feature Sets and Kernel Functions

The use of Multiple Kernel Learning gave us the opportunity to not only select a large number of heterogeneous features, but also assign a specifically adapted kernel function to each set. Through review of biological hypotheses and preliminary results we identified a number of feature types and kernel functions most likely to yield good performance for cleavage prediction. We were then able to run several different configurations in order to evaluate which combination produced the best compromise between performance and model complexity.

In addition to the classic Gaussian Radial Basis Function (RBF) kernel (commonly used on such problem, in conjunction with binary-encoded vectors of amino acid positions; see for example [38]), we examined two other types of kernel functions that offered interesting alternative perspectives on our data:

#### String kernel

Similar to linear or RBF kernel functions, in that it is position-dependent, a typical string kernel function calculates the number of identical *k*-mers (of length varying between 1 and the kernel order: 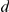) between two sequences of length 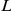 and can be defined as:
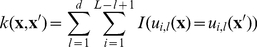
(4)


Where 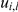 refers to the substring of 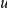 of length 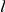 starting at position 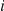, and 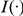 is the indicator function.

It offers the advantage of working directly on string data (removing the need for binary encoding of sequences and leading to more compact feature vectors) and can be configured to look at *k*-mers instead of being restricted to single amino acid position in a sequence. These two aspects make it well-suited to examine position-based sequence features.

#### Spectrum kernel

Spectrum kernels are a family of functions based on position-independent *k*-mer enumerations. In this instance, we use gapped substring kernels, defined as:
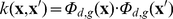
(5)


Where 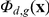 returns a vector of occurrence counts for all k-mers of length at most 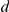 and allowing for at most 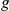 gaps within string 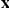.

Using this type of kernel function lets us focus on the search for feature motifs anywhere in the sub-sequence, regardless of position or window size. It is therefore particularly adapted for structural features, such as secondary structure or solvent accessibility: accommodating their typically flexible nature by allowing for looser positioning around the cleavage site.

### Using Calpain Type Specificity

**Figure 1:**
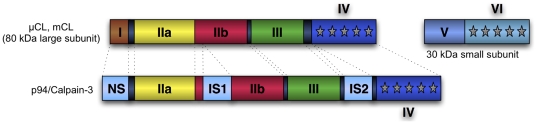
Schematic structures of major calpain homologues. “Conventional” calpains (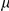
- and m-calpain) are composed of larger catalytic subunits (calpain-1 and -2) and a smaller regulatory subunit. Some homologues, such as skeletal muscle-specific calpain (calpain-3/p94) have slightly diverged properties, including unique insertion sequences (NS, IS1 and IS2) and no requirement for a small subunit. Symbols used are: **I**: N-terminal domain with little homology; **IIa** and **IIb**: protease sub-domains containing the active sites Cys and His/Asn, respectively; **III**: C2-like 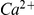-binding domain; **IV** and **VI**: 5-EF-hand 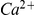-binding domain; **V**: Gly-rich hydrophobic domain; **NS**, **IS1** and **IS2**: p94-specific sequences.

Humans present 15 genes that encode a calpain-like protease domain, generating diverse kinds of calpain homologues with combinations of several functional domains such as 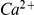-binding domains (C2-domain-type and EF-hand-type) and Zn-finger domains. Additionally, calpain homologues are increasingly being found in other organisms including insect, nematode, trypanosome, plant, fungus, yeast and even some bacteria. The substrates present in our data (Figure 1) were mainly shared between 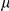-calpain (heterodimer of calpain-1 and CAPNS1) and m-calpain (heterodimer of calpain-2 and CAPNS1), two major ubiquitous homologues activated respectively by 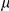
M and mM levels of 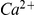 concentrations *in vitro*. In addition to these two types, a muscle-specific calpain known as calpain-3 (also called p94) accounted for two more substrate sequences.

**Figure 2:**
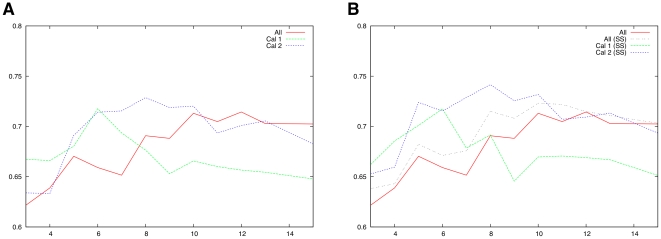
Linear-kernel SVM performance trained on full set of substrates (All) vs. calpain-1 (Cal 1) and calpain-2 (Cal 2). AUC score as function of symetrical extension length (number of nucleotides) on each side of putative cleavage site. **A**: using only position information. **B**: using position and secondary structure (SS) information.

While it is generally considered that members of the calpain family behave similarly in their proteolytic activity [1], [42], [43], preliminary results (Figure 2) showed that some amount of specificity may exist with regard to substrate sequence and cleavage location. Despite potential issues with reducing the amount of training data even further, we investigated the hypothesis that separating data by calpain type might lead to improved prediction quality.

### Experimental Setting

The data used in all our experiments was obtained from the online calpain database CaMPDB [37], selecting only confirmed substrate sequences (“SB” label). The issue of selection bias in the curated set was addressed by removing redundant sequences (as defined by presenting an alignment with over 95% identity to another sequence in the set) resulting in a set of 90 sequences.

For each sequence, we computed secondary structure and solvent accessibility data using, respectively, PSIPRED [44] and ACCpro [45]. Each amino acid sequence was thus labeled with 3 classes for secondary structure (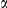-helix, 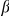-sheet, “other”) and two classes for solvent accessibility (above and below a 25% accessibility treshold).

The different kernels were trained and evaluated using the Shogun framework [39] through its Python modular interface. Windows of varying sizes around each cleavage site made the set of positive instances, while negative instances where randomly sampled from every other position in the sequence so as to yield a 10 to 1 ratio between negative and positive instances.

All performance results were measured using Area under ROC Curve (AUC) with 10×10 cross-validation (average of all AUC values generated from 10 repeats of 10-fold cross-validation).

When used with Gaussian or Linear kernels, all feature vectors were extracted from sequence data using a canonical binary encoding (each amino acid position in the primary sequence was matched by 20 binary values in the feature vector). Raw sequence data was used as input for string and spectrum kernels.

Optimal SVM parameters (cost: 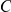 and, where applicable, kernel width: 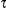) were set for each kernel using a grid search (see Table 1).

**Table 1:** SVM Parameters.

Parameter	Min	Max	Optimal Value
**C (cost)**	0.1	10	1.67
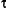 **(width)**	.001	1000	2.1
**d (order of string kernel)**	1	6	5
**d**' **(order of spectrum kernel)**	1	10	8
**g (gaps allowed)**	0	3	1

Tested range and optimal values for SVM kernel function parameters. Integer values were tested for the entire range. Non-integer parameters were set using values within their ranges in two successive grid search of decreasing step value.

For each combined kernel the optimal window-length parameters (left and right extension around cleavage site position) of each sub-kernel were found by sequentially running a grid search on one set of parameters while freezing the others. This process was iterated until convergence of the top cross-validated AUC score, yielding locally optimal parameters (between 2 and 8, depending on kernel configuration) in reasonable computational time.

We first explored the impact of calpain-type specificity by running limited cross-validation experiments on the full set of substrate sequences, then on two subsets made of substrates cleaved by calpain-1 and calpain-2 respectively (the set of substrates cleaved by calpain-3 was too small to be efficiently analyzed and was therefore ignored in this part).

As a baseline we computed optimal AUC scores using a single Gaussian kernel: first on sequence data alone, then on sequence and secondary structure concatenated together using encoding and window length parameters described in [38] and finally on a variant of [38] using a grid search to find optimal extension length parameters.

Finally, AUCs were computed for three configurations of combined kernel, using the method developed by [39] to simultaneously optimize sub-kernel weights and matrices.

## Results and Discussion

### Preliminary Results

We analyzed the impact of using different extension sizes on either side of cleavage sites. In particular, we looked for pronounced asymmetrical features. In order to keep the size of input features down and avoid unnecessary noise, it was critical to accurately narrow down sequence regions directly or indirectly involved in substrate recognition and cleavage for each type of feature (sequence, secondary structure and solvent accessibility).

When comparing single-kernel performances across calpain-type (Figure 2) we can observe that, while AUC performance peaks at 6 amino acids around the cleavage site for the 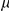-calpain (Cal-1) set, performance on the m-calpain (Cal-2) set increases until at least the 8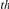 amino acid.

**Figure 3:**
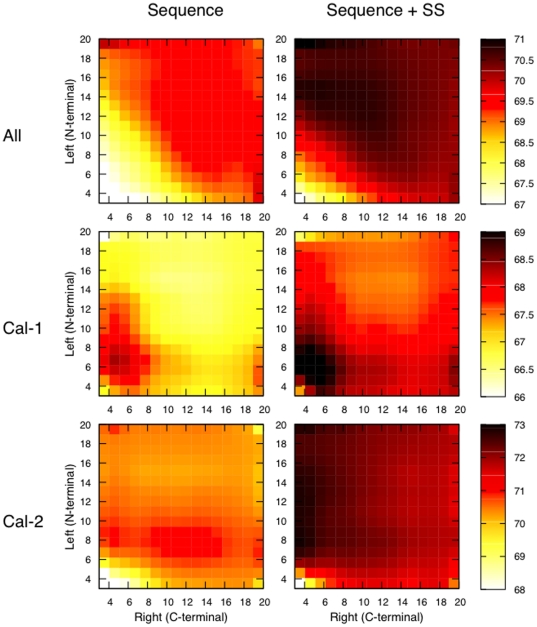
AUC (with a linear-kernel SVM) as function of cleavage extension length (left and right side of cleavage site) in number of nucleotides. Left column uses sequence only, while right column uses secondary structure information (SS) as well.

**Figure 4:**
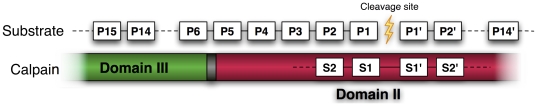
Schematic representation of contact region between calpain and substrate sequence. Domain II is the protease domain of calpain, while domain III binds 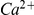. Amino acid sequences of domain III are less conserved than those of domain II, which are highly conserved not only between 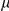
- and m-calpains but also among all calpain family members.

This trend is even more visible when considering asymmetrical extension lengths (Figure 3), where we can clearly see important differences between 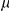-calpain and m-calpain. Along the Y-axis, for 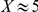 (that is, with P1′–P5′ fixed and extending toward P1, P2, P3...), 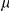-calpain has a peak around 6 and quickly decays after that. On the other hand, m-calpain predictions perform well until around the 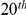 amino acid. This probably indicates that 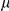-calpain recognizes a relatively short stretch of the N-terminal side of substrates (until around P6), whereas m-calpain uses longer portion of the N-terminal side of substrates (P20 and beyond). In other words, 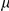-calpain probably recognizes substrates mainly by domain II (Figure 4), while m-calpain also uses domain III for recognition of (at least some) substrates. This may indicate that 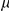-calpain has more affinity to short peptides than m-calpain. Considering that 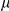
- and m-calpain have similar catalytic velocity (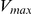), this would imply that 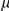-calpain has larger turn-over numbers for short peptides. While in our own experience (unpublished work) 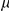-calpain has indeed showed higher activity to peptide substrates than m-calpain (over 5-fold), current literature presents arguments both partly in favor [46] and against [47] this hypothesis.

One interesting difference was reported from 3D structural studies: when the protease domain (subdomains IIa and IIb, Figure 1) from either type of calpain was isolated, expressed and used for proteolytic assay, the domain from 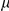-calpain showed over 1000-fold more activity than m-calpain. Structurally, this phenomenon could be explained by interferences of the active site with Trp106, due to the lack of interaction between subdomain IIa and domain III resulting in instability of the Gly197–Gly210 loop [48]. In contrast, whole 3D structure of m-calpain, composed of domains I−IV+VI, showed stabilized Gly197–Gly210 loop and no interference with Trp106 [49].

It is possible that some substrates may interact with domain III of m-calpain, resulting in disruption of the interaction between Gly197–Gly210 loop and domain III, which would interfere with the interaction between domain IIa's S2–S3 sites and substrates (with corresponding P2–P3 residues). In this case, m-calpain would use over S4 sites for substrate recognition, which may explain the difference we observed between m- and 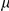-calpain.

In all cases (Cal-1, Cal-2 and ‘All’), the addition of secondary structure information (SS) to the linear kernel's input features, not only resulted in higher AUC across the board but also attenuated the previously observed impact of features length specificity within each calpain subset (Figure 3, right column). However, it is interesting to note that this attenuation is less pronounced for 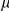-calpain (Cal-1) than for m-calpain (Cal-2): this may indicate that m-calpain has stronger substrate-specificity at the secondary structure level than 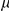-calpain. There is no experimental evidence that may support this hypothesis but it is consistent with the previously mentioned specificity of m-calpain over wider substrate areas (since secondary structure only makes sense for oligopeptides of about 10 residues or more).

Finally, there is an imprecise but significant “line” along the X-axis at 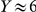 for both 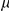
- and m-calpain. This may indicate that, if substrates do not interact well with domain III (i.e. P6–P14), they interact more with domain II via P6′–P14′, implying that calpain use different ranges of its structure to recognize different substrates: a possible explanation for calpain's ability to recognize such a variety of substrates with a single molecule.

When switching to a non-linear single kernel (Gaussian RBF), performance increased significantly (Table 2). However, in contrast to the linear model results and despite previous findings [38], the addition of secondary structure information not only failed to bring significant improvement but, in most cases lowered AUC results (Table 2, I′ and I′′). This performance hit could be caused by the extra noise brought by the addition of overly rigid position-specific secondary structure information, compounded by the high dimension and sparsity of the resulting feature space: a type of problem often better handled by simpler linear model over complex kernels (although the added discriminative power of the non-linear model still results in overall better performance on sequence alone). The unsatisfying compromise of having to choose a single kernel and common encoding for both types of information further hinted at the potential benefit of our multiple kernel approach. Interestingly, AUC results for the calpain-2 subset showed much better resistance (if no significant improvement) to the addition of secondary structure features: a further confirmation that specificity by calpain type might exist, with at least different use of secondary structure information.

**Table 2:** AUC Results with single Gaussian kernel methods.

	Cal 1	Cal 2	All
**Position (I)**	77.77 (0.88)	77.09 (1.28)	76.86 (1.05)
**Position + SS** * **(I**′**)**	73.25 (2.00)	74.13 (1.57)	75.39 (1.11)
**Position + SS** ** **(I**′′**)**	73.25 (2.00)	77.22 (1.19)	75.39 (1.11)

*: using same encoding and window length as [38].

**: using same encoding as [38] but with optimal window parameters obtained through grid search.

Results are shown as: % AUC (% SEM).

**Position**: Residue position information, with a Gaussian RBF kernel (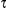 = 2.1, 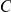 = 1.67) and canonical binary encoding. **SS**: Secondary structure, with a Spectrum kernel (*k* between 2 and 5, allowing up to 1 gap).

### Multiple Kernel Learning Results

We obtained a top AUC score of 83.36% on the full training set (‘All’), using MKL with a combined kernel containing position, string and secondary structure information (Table 3). Despite using no more input data (sequence and secondary structure) than single kernel methods (Table 2, I′′), our method resulted in a considerable AUC increase from the baseline score of 76.86% (pairwise T-test p-value 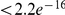, between 10×10 cross-validation AUC results, with equal variance assumption). Although the addition of solvent accessibility seemed to improve scores, the increase was not significant compared to the introduction of secondary structure alone (Table 4).

**Table 3:** AUC Results with MKL methods.

	Cal 1	Cal 2	All
**String + SS (II)**	82.39 (0.70)	80.20 (0.67)	81.46 (0.54)
**Position + String + SS (III)**	84.28 (0.67)	83.09 (0.79)	83.36 (0.54)
**Position + String + SS + SA (IV)**	85.46 (0.66)	83.23 (0.68)	83.71 (0.59)

In addition to legends from table 2:

**String**: Sequence string, with a String kernel (position-based substrings of length 1 to 6). **SA**: 25% solvent accessibility, with a Spectrum kernel (*k* between 1 and 7, allowing up to 1 gap).

**Table 4:** Pairwise T-test Comparison.

Test	p-value	Conclusion
(I) vs. (II)	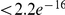	Significant
(II) vs. (III)	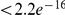	Significant
(III) vs. (IV)	0.6029	Inconclusive
(II) vs. (IV)	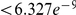	Significant

P-values for pairwise T-test comparisons between results from different combination of kernels, using sets of 10×10 AUC results, under assumption of equal variance.

Due to the limited availability of cleavage data (less than 90 distinct sequences for all calpain types, see Table 5), it could be expected that breaking down the general set into smaller calpain gene product subsets (of approximately half the size) would hurt performances: yet, results were not only stable within each subset, but in some cases, improved. Interestingly, solvent accessibility brought a significantly higher score to the calpain-1 subset (85.46%), indicating that the mechanisms of substrate recognition by 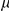-calpain might rely more heavily on this property than m-calpain.

**Table 5:** Substrate labeling by calpain sub-type.

	Calpain-1	Calpain-2	Calpain-3	All Types
Substrate sequences	46	49	2	90
Cleavage sites	94	114	4	220

Values from each calpain gene types do not add up to the figures for ‘All Types’, due to some substrates being cleaved by more than one type, while other sequences are missing calpain type labeling.

**Figure 5:**
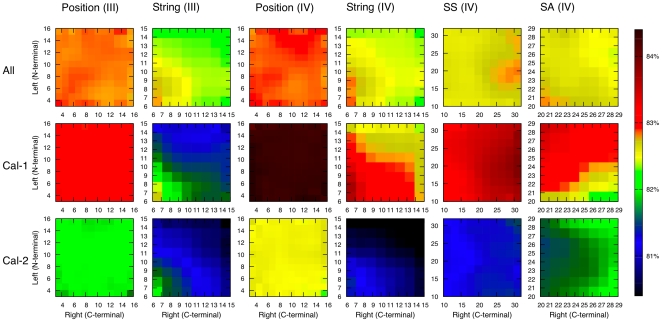
AUC as function of cleavage extension length. AUC values produced by MKL prediction method, when varying extension length for one feature set at a time (all other parameters at their optimal value). See table 2 and 3 for notations.

In most instances, optimal window length parameters showed a strong asymmetry between left- and right-side extension around cleavage site (Figure 5).

Analyzing the final weights (Table 6) for each sub-kernel in the MKL method (computed on normalized kernel matrices), we were able to confirm what raw AUC results (Table 3) plainly suggested in terms of feature selection: similar orders of magnitude between the weight for position-based features and those for secondary structure and solvent accessibility, can be formally interpreted as an indication that the latter still bring discriminative power to the combined classifier [39].

**Table 6:** MKL weights.

	Position	String	SS	SA
**String + SS (II)**	-	1.0	0.09	-
**Position + String + SS (III)**	0.80	0.59	0.07	-
**Position + String + SS + SA (IV)**	0.78	0.59	0.06	0.18

Optimal training weights obtained for each combination of kernels (on full calpain set) using MKL training algorithm described in [39].

### Validation with Mutant Calpastatin Sequences

Calpastatin is an endogenous inhibitor protein specific to calpain [50], [51]: after activation by 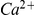, calpain is recognized by calpastatin, which binds to its active site while remaining uncleaved, thus inhibiting proteolytic activity. In their experimental work, Moldoveanu *et al.* were able to induce proteolysis in several mutant sequences of calpastatin by deleting one or two specific residues (Lys176, Glu177, or both) from a sequence of wild type rat calpastatin (gi 13540322) [49]. Both sequences of calpastatin (wild type and mutants) being phylogenetically unrelated to all substrate sequences in our training set, they provided a good opportunity for qualitative validation on the generalization power of our prediction method.

**Figure 6:**
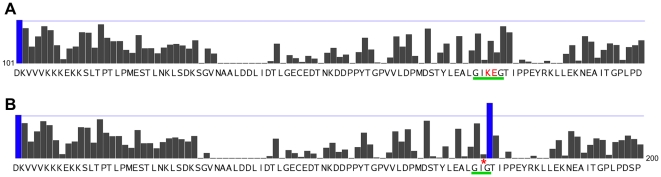
Cleavage prediction on Calpastatin sequences. Normalized MKL prediction scores using Position, String and SS feature sets. **A**: on wild type *Rattus norvegicus* calpastatin (gi 13540322). **B**: on a mutant of calpastatin, obtained by deletion of Lys176 and Glu177 (highlighted in red in sequence **A** and marked by a red star in sequence **B**). Results were cropped to residues [101–200] in the sequence. Thin blue line marks 5% top scores threshold. Thick green lines highlight “loop-out” area of calpastatin sequences (shortened in the mutant by deletion of Lys176 and Glu177) where cleavage would likely occur.

The 3D structure of co-crystallized m-calpain and calpastatin [49], [52] indicated that Leu172-Gly173 and Thr179-Ile180 are at the P2–P1 and P1′–P2′ positions, respectively. Deletion of Lys176 and Glu177 makes this mutant calpastatin a good substrate, strongly suggesting, in the absence of further experimental confirmation, that the cleavage site is at the C-terminus of Ile175 and/or Gly178. Indeed, our program predicted a sharp peak between Gly178 and Thr179 for this mutant calpastatin (Figure 6B). The results presented in figure 6 showed not only that our prediction model correctly identifies the binding site in the original calpastatin sequence as a poor candidate for cleavage, but most importantly, detected a sharp signal increase on the same site after the sequence had been altered to allow cleavage by calpain, closely matching what has been experimentally observed by Moldoveanu *et al.* [49].

### Conclusion

Through the use of a novel extension to the classic SVM framework, we were able to significantly improve cleavage prediction performance, as measured by a critical AUC increase: from less than 77% (RBF position-based score for the full calpain set) to over 83% (combined kernel using secondary structure on top of sequence information). The demonstrated inability of single-kernel methods to benefit from the addition of extra features such as secondary structure, presumed to be helpful [28], provides a strong argument in favor of MKL: by allowing seamless integration of heterogeneous features while retaining their respective structure, MKL can yield satisfying performance on even critically small training sets.

Furthermore, we presented results strongly favoring the hypothesis that subtypes of calpain behave differently with regard to substrate recognition and cleavage, dispelling previous assumption that proteolytic action was identical across all types of calpain (treating subtypes separately lead to significant performance increase in the case 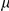-calpain where AUC was improved by a further 2% to 85.46%).

In future work, we plan to explore the possibility of adapting this method to general cleavage prediction for other types of proteases (such as proteasomes). Additionally, the recently suggested use of generalized 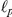-norm (for values of 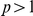) to promote weight sparsity [53] could allow us to consider much greater combinations of kernels at a time while preserving model accuracy.

An online implementation of the prediction method presented in this article is available at http://calpain.org.
